# Poly[[(*N*,*N*-dimethyl­formamide-κ*O*)(μ_3_-pyrazine-2,3-dicarboxyl­ato-κ^4^ *N* ^1^,O^2^:*O* ^3^:*O* ^3^)copper(II)] monohydrate]

**DOI:** 10.1107/S1600536809051332

**Published:** 2009-12-04

**Authors:** Zhen-Zhong Fan, Guo-Ping Wang, Yu-Sheng Li

**Affiliations:** a Department of Petroleum Engineering, Daqing Petroleum Institute, Heilongjiang 151400, People’s Republic of China; b Department of Chemistry, Zhejiang University, People’s Republic of China; c Second Oil Recovery Plant, Daqing Oilfields Co, Daqing 163414, People’s Republic of China

## Abstract

In the title compound, {[Cu(C_6_H_2_N_2_O_4_)(C_3_H_7_NO)]·H_2_O}_*n*_, the Cu(II) atom is coordinated by an *N,O*-bidentate pyrazine-2,3-dicarboxyl­ate (pzdc) dianion, two O atoms from two other pzdc anions and one O atom from the dimethlyformamide ligand, forming a distorted square-pyramidal CuNO_4_ geometry. The polymeric character of the structure is established by the formation of layers parallel to (100) *via* bridging pzdc ligands. O—H⋯O hydrogen bonding between water mol­ecules and uncoordinated carboxyl­ate O atoms leads to additional stabilization of the structure.

## Related literature

For related structures with the pyrazine-2,3-dicarboxyl­ate (pzdc) dianion, see: Hua & Liu (2009 ▶); Konar *et al.* (2004 ▶); Li *et al.* (2004 ▶); Lin *et al.* (2009 ▶); Tombul & Guven (2009 ▶); Wang *et al.* (2008 ▶); Xiang *et al.* (2004 ▶); Xu *et al.* (2008 ▶).
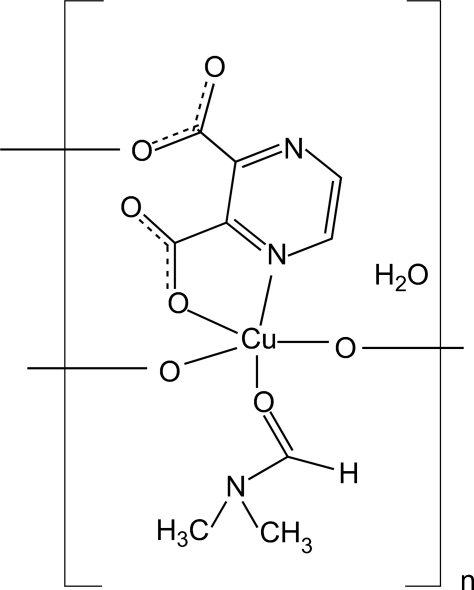


## Experimental

### 

#### Crystal data

[Cu(C_6_H_2_N_2_O_4_)(C_3_H_7_NO)]·H_2_O
*M* *_r_* = 320.75
Monoclinic, 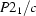

*a* = 10.1656 (5) Å
*b* = 13.6310 (8) Å
*c* = 9.1461 (2) Å
β = 91.430 (2)°
*V* = 1266.96 (10) Å^3^
*Z* = 4
Mo *K*α radiation
μ = 1.75 mm^−1^
*T* = 298 K
0.39 × 0.10 × 0.06 mm

#### Data collection

Bruker APEX area-detector diffractometer
Absorption correction: integration (*SADABS*; Bruker, 2002 ▶) *T*_min_ = 0.549, *T*_max_ = 0.902
6222 measured reflections
2283 independent reflections
1600 reflections with *I* > 2σ(*I*)
*R*_int_ = 0.058

#### Refinement

*R*[*F* ^2^ > 2σ(*F* ^2^)] = 0.051
*wR*(*F* ^2^) = 0.115
*S* = 0.98
2283 reflections
180 parameters
3 restraints
H atoms treated by a mixture of independent and constrained refinement
Δρ_max_ = 0.48 e Å^−3^
Δρ_min_ = −0.34 e Å^−3^

### 

Data collection: *SMART* (Bruker, 2002 ▶); cell refinement: *SAINT* (Bruker, 2002 ▶); data reduction: *SAINT*; program(s) used to solve structure: *SHELXS97* (Sheldrick, 2008 ▶); program(s) used to refine structure: *SHELXL97* (Sheldrick, 2008 ▶); molecular graphics: *SHELXTL* (Sheldrick, 2008 ▶); software used to prepare material for publication: *SHELXTL*.

## Supplementary Material

Crystal structure: contains datablocks I, global. DOI: 10.1107/S1600536809051332/wm2283sup1.cif

Structure factors: contains datablocks I. DOI: 10.1107/S1600536809051332/wm2283Isup2.hkl

Additional supplementary materials:  crystallographic information; 3D view; checkCIF report

## Figures and Tables

**Table 1:** Hydrogen-bond geometry (Å, °)

*D*—H⋯*A*	*D*—H	H⋯*A*	*D*⋯*A*	*D*—H⋯*A*
O6—H6*B*⋯O4^i^	0.82 (5)	2.00 (3)	2.771 (5)	156 (7)
O6—H6*A*⋯O1^ii^	0.82 (5)	2.38 (3)	3.138 (6)	153 (5)
O6—H6*A*⋯O2^ii^	0.82 (5)	2.30 (3)	3.037 (5)	149 (5)

Symmetry codes: (i) 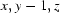; (ii) 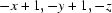.

**Fig. 1.**
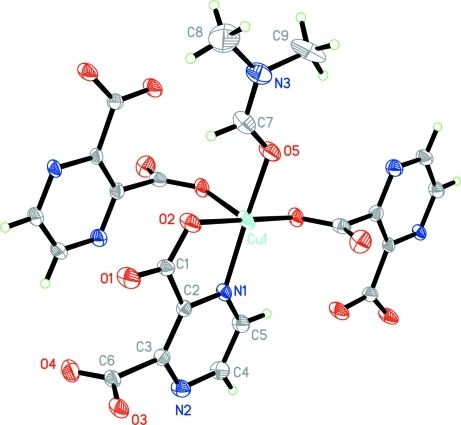
The coordination of the Cu(II) atom in the structure of compound (I), showing atom labels and 50% probability displacement ellipsoids for the non-H atoms.

**Fig. 2.**
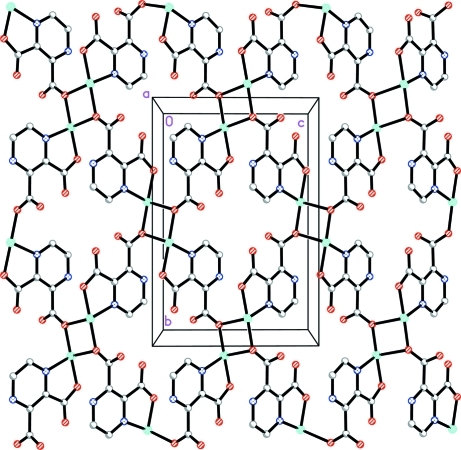
The polymeric structure of compound (I); water molecules and DMF have been omitted for clarity.

## supplementary crystallographic information

### Comment

Polymeric compounds play an important role in the field of molecular magnetism. Di- and polycarboxylates are capable of bridging and providing effective magnetic exchange pathways. Pyrazine-2,3-dicarboxylic acid (H_2_pzdc) has been widely applied to construct polymeric coordination compounds; their structures and magnetic properties were investigated (Li *et al.*, 2004; Xiang *et al.*, 2004; Hua & Liu, 2009; Lin *et al.*, 2009; Tombul & Guven, 2009). We present here the structure of the title compound, {[Cu(pzdc)(DMF)]^.^H_2_O}_n_, (I).

In the structure of compound (I), each Cu(II) atom is coordinated by an N atom of the pyrazine ring and a carboxylic O atom from one pzdc^2-^ anion, two O atoms from two other pzdc^2-^ anions, and O atom from DMF, forming a distorted square-pyramidal environment, as depicted in Fig. 1. The four atoms N1, O2, O5 and O3^i^ form the basal plane, whereas the O3^ii^ atom [symmetry code: (i) -*x* +1, *y* - 1/2, -*z* + 1; (ii) *x*, -*y* + 3/2, *z* -1/2] occupies the apical site with a longer Cu1—O3^ii^ bond length of 2.378 (3) /%A. In the basal plane, the bond distances of Cu1—N1 and Cu1—O3^i^ are 2.002 (3) and 1.958 (3) Å, which are shorter than those observed for the distorted square-pyramidal CuN_2_O_3_ coordination in a related structure of a Cu(II)pzdc derivative (Hua & Liu, 2009). The plane defined by carboxylic group O1—C1—C2—C3 is nearly coplanar to the pyrazine ring with the dihedral angle of 3.7 (7)°, while another carboxylate plane defined by the C2—C3—C6—O4 is approximately perpendicular to the pyrazine ring [dihedral angel of 92.9 (5)°]. This conformation was also observed in a series of other transition metal complexes formed with H_2_pzdc (Li *et al.*, 2004; Xiang *et al.*, 2004; Konar *et al.*, 2004; Hua *et al.*, 2009). Extensive hydrogen bonding interactions help to stabilize the structure. The carboxylate O atoms take part in a O—H···O hydrogen bonding and are acceptor atoms with O atoms of water molecules as donator atoms (Table 1).

### Experimental

Mixed DMF and aqueous solution (15 ml) of CuCl_2_^.^2H_2_O (0.5 mmol) and 2,3-pyrazinedicarboxylic acid ((H_2_pzdc, 0.25 mmol) were slowly added into a methanolic (5 ml) solution of triethylene diamine (TED, 0.5 mmol); the resulting mixture was stirred for 5 minutes and allowed to stand at room temperature for about four days until blue single crystals were obtained.

### Refinement

H atoms of the water molecules were located in a difference Fourier map and were refined isotropically, with O—H and H—H distance restraints of 0.84 (1) Å and 1.37 (2) Å, respectively. The remaining H atoms were positioned geometrically (C—H = 0.93 Å) and allowed to ride on their parent atoms. The *U*_iso_(H) values were set at 1.2*U*_eq_(C) and 1.5*U*_eq_(O).

### Crystal data

**Table:**

[Cu(C_6_H_2_N_2_O_4_)(C_3_H_7_NO)]·H_2_O	*F*(000) = 652
*M**_r_* = 320.75	*D*_x_ = 1.682 Mg m^−^^3^
Monoclinic, *P*2_1_/*c*	Mo *K*α radiation, λ = 0.71073 Å
Hall symbol: -P 2ybc	Cell parameters from 1111 reflections
*a* = 10.1656 (5) Å	θ = 2.5–22.4°
*b* = 13.6310 (8) Å	µ = 1.75 mm^−^^1^
*c* = 9.1461 (2) Å	*T* = 298 K
β = 91.430 (2)°	Prism, blue
*V* = 1266.96 (10) Å^3^	0.39 × 0.10 × 0.06 mm
*Z* = 4	

### Data collection

**Table:**

Bruker APEX area-detector diffractometer	2283 independent reflections
Radiation source: fine-focus sealed tube	1600 reflections with *I* > 2σ(*I*)
graphite	*R*_int_ = 0.058
phi and ω scans	θ_max_ = 25.3°, θ_min_ = 2.0°
Absorption correction: integration (*SADABS*; Bruker, 2002)	*h* = −8→12
*T*_min_ = 0.549, *T*_max_ = 0.902	*k* = −16→16
6222 measured reflections	*l* = −10→10

### Refinement

**Table:**

Refinement on *F*^2^	Primary atom site location: structure-invariant direct methods
Least-squares matrix: full	Secondary atom site location: difference Fourier map
*R*[*F*^2^ > 2σ(*F*^2^)] = 0.051	Hydrogen site location: inferred from neighbouring sites
*wR*(*F*^2^) = 0.115	H atoms treated by a mixture of independent and constrained refinement
*S* = 0.98	*w* = 1/[σ^2^(*F*_o_^2^) + (0.0482*P*)^2^] where *P* = (*F*_o_^2^ + 2*F*_c_^2^)/3
2283 reflections	(Δ/σ)_max_ < 0.001
180 parameters	Δρ_max_ = 0.48 e Å^−^^3^
3 restraints	Δρ_min_ = −0.33 e Å^−^^3^

### Special details

**Table:**

Geometry. All e.s.d.'s (except the e.s.d. in the dihedral angle between two l.s. planes) are estimated using the full covariance matrix. The cell e.s.d.'s are taken into account individually in the estimation of e.s.d.'s in distances, angles and torsion angles; correlations between e.s.d.'s in cell parameters are only used when they are defined by crystal symmetry. An approximate (isotropic) treatment of cell e.s.d.'s is used for estimating e.s.d.'s involving l.s. planes.
Refinement. Refinement of *F*^2^ against ALL reflections. The weighted *R*-factor *wR* and goodness of fit *S* are based on *F*^2^, conventional *R*-factors *R* are based on *F*, with *F* set to zero for negative *F*^2^. The threshold expression of *F*^2^ > σ(*F*^2^) is used only for calculating *R*-factors(gt) *etc*. and is not relevant to the choice of reflections for refinement. *R*-factors based on *F*^2^ are statistically about twice as large as those based on *F*, and *R*- factors based on ALL data will be even larger.

### Fractional atomic coordinates and isotropic or equivalent isotropic displacement parameters (Å^2^)

**Table:**

	*x*	*y*	*z*	*U*_iso_*/*U*_eq_	
Cu1	0.59999 (5)	0.58784 (3)	0.07521 (6)	0.0402 (2)	
O1	0.5775 (3)	0.8747 (2)	0.1085 (4)	0.0526 (10)	
O2	0.6295 (3)	0.7254 (2)	0.0382 (4)	0.0463 (9)	
O3	0.4380 (3)	0.9489 (2)	0.3914 (3)	0.0386 (8)	
O4	0.2821 (3)	0.9362 (2)	0.2189 (4)	0.0511 (9)	
O5	0.7494 (3)	0.5510 (2)	−0.0435 (4)	0.0503 (9)	
O6	0.2218 (5)	0.1287 (3)	0.1467 (7)	0.121 (2)	
N1	0.4710 (3)	0.6417 (2)	0.2162 (4)	0.0298 (8)	
N2	0.3001 (3)	0.7442 (2)	0.3914 (4)	0.0402 (9)	
N3	0.9066 (4)	0.5894 (4)	−0.2048 (5)	0.0655 (13)	
C1	0.5654 (4)	0.7859 (3)	0.1145 (5)	0.0331 (10)	
C2	0.4684 (4)	0.7404 (3)	0.2158 (4)	0.0279 (9)	
C3	0.3821 (4)	0.7913 (3)	0.3018 (5)	0.0316 (10)	
C4	0.3064 (4)	0.6467 (3)	0.3912 (5)	0.0420 (12)	
H4	0.2516	0.6118	0.4523	0.050*	
C5	0.3912 (4)	0.5951 (3)	0.3039 (5)	0.0381 (11)	
H5	0.3920	0.5269	0.3071	0.046*	
C6	0.3670 (4)	0.9018 (3)	0.3005 (5)	0.0352 (10)	
C7	0.8054 (5)	0.6097 (4)	−0.1238 (6)	0.0554 (14)	
H7	0.7732	0.6735	−0.1271	0.066*	
C8	0.9664 (6)	0.6630 (5)	−0.2983 (8)	0.112 (3)	
H8A	0.9216	0.7244	−0.2874	0.168*	
H8B	0.9593	0.6421	−0.3985	0.168*	
H8C	1.0575	0.6707	−0.2706	0.168*	
C9	0.9607 (6)	0.4924 (5)	−0.2062 (7)	0.092 (2)	
H9A	1.0486	0.4937	−0.1655	0.137*	
H9B	0.9624	0.4688	−0.3051	0.137*	
H9C	0.9073	0.4496	−0.1491	0.137*	
H6A	0.277 (5)	0.148 (4)	0.089 (6)	0.110*	
H6B	0.219 (6)	0.0685 (9)	0.155 (7)	0.110*	

### Atomic displacement parameters (Å^2^)

**Table:**

	*U*^11^	*U*^22^	*U*^33^	*U*^12^	*U*^13^	*U*^23^
Cu1	0.0472 (4)	0.0182 (3)	0.0560 (4)	0.0032 (2)	0.0188 (3)	0.0026 (3)
O1	0.075 (2)	0.0191 (15)	0.065 (3)	−0.0083 (15)	0.029 (2)	−0.0011 (16)
O2	0.0559 (19)	0.0221 (15)	0.062 (2)	−0.0013 (14)	0.0282 (17)	0.0013 (16)
O3	0.0467 (17)	0.0189 (14)	0.050 (2)	−0.0034 (13)	0.0072 (16)	−0.0062 (15)
O4	0.060 (2)	0.0361 (18)	0.057 (2)	0.0112 (16)	−0.0027 (19)	−0.0003 (17)
O5	0.047 (2)	0.0345 (17)	0.070 (3)	0.0050 (15)	0.0268 (18)	0.0034 (17)
O6	0.102 (4)	0.071 (3)	0.192 (6)	0.010 (3)	0.078 (4)	0.054 (4)
N1	0.0353 (19)	0.0157 (17)	0.039 (2)	−0.0021 (14)	0.0026 (17)	0.0003 (16)
N2	0.049 (2)	0.029 (2)	0.043 (2)	−0.0043 (17)	0.0103 (19)	−0.0032 (18)
N3	0.041 (2)	0.083 (3)	0.074 (4)	0.010 (2)	0.021 (2)	0.004 (3)
C1	0.040 (2)	0.025 (2)	0.034 (3)	−0.0004 (19)	0.008 (2)	−0.002 (2)
C2	0.036 (2)	0.019 (2)	0.029 (3)	−0.0042 (17)	−0.0021 (19)	−0.0008 (18)
C3	0.038 (2)	0.025 (2)	0.032 (3)	−0.0013 (18)	−0.001 (2)	−0.0019 (19)
C4	0.052 (3)	0.030 (2)	0.045 (3)	−0.010 (2)	0.011 (2)	0.002 (2)
C5	0.050 (3)	0.021 (2)	0.044 (3)	−0.004 (2)	0.007 (2)	0.000 (2)
C6	0.039 (2)	0.026 (2)	0.041 (3)	0.004 (2)	0.014 (2)	0.004 (2)
C7	0.043 (3)	0.053 (3)	0.070 (4)	0.009 (2)	0.005 (3)	−0.007 (3)
C8	0.072 (4)	0.144 (7)	0.121 (7)	0.014 (4)	0.049 (4)	0.053 (5)
C9	0.062 (4)	0.098 (5)	0.116 (6)	0.011 (4)	0.030 (4)	−0.050 (4)

### Geometric parameters (Å, °)

**Table:**

Cu1—O2	1.930 (3)	N2—C3	1.346 (5)
Cu1—O5	1.954 (3)	N3—C7	1.313 (6)
Cu1—O3^i^	1.958 (3)	N3—C9	1.432 (6)
Cu1—N1	2.002 (3)	N3—C8	1.460 (7)
Cu1—O3^ii^	2.378 (3)	C1—C2	1.504 (5)
O1—C1	1.218 (5)	C2—C3	1.380 (5)
O2—C1	1.271 (5)	C3—C6	1.515 (5)
O3—C6	1.263 (5)	C4—C5	1.381 (6)
O3—Cu1^iii^	1.958 (3)	C4—H4	0.9300
O3—Cu1^iv^	2.378 (3)	C5—H5	0.9300
O4—C6	1.220 (5)	C7—H7	0.9300
O5—C7	1.235 (6)	C8—H8A	0.9600
O6—H6A	0.82 (5)	C8—H8B	0.9600
O6—H6B	0.82 (5)	C8—H8C	0.9600
N1—C5	1.318 (5)	C9—H9A	0.9600
N1—C2	1.344 (4)	C9—H9B	0.9600
N2—C4	1.331 (5)	C9—H9C	0.9600
			
O2—Cu1—O5	91.48 (13)	C3—C2—C1	125.4 (4)
O2—Cu1—O3^i^	177.39 (13)	N2—C3—C2	121.3 (4)
O5—Cu1—O3^i^	89.82 (13)	N2—C3—C6	114.5 (4)
O2—Cu1—N1	82.17 (12)	C2—C3—C6	124.2 (4)
O5—Cu1—N1	169.37 (13)	N2—C4—C5	122.6 (4)
O3^i^—Cu1—N1	96.87 (12)	N2—C4—H4	118.7
O2—Cu1—O3^ii^	100.87 (12)	C5—C4—H4	118.7
O5—Cu1—O3^ii^	94.99 (12)	N1—C5—C4	120.6 (4)
O3^i^—Cu1—O3^ii^	76.75 (12)	N1—C5—H5	119.7
N1—Cu1—O3^ii^	94.57 (12)	C4—C5—H5	119.7
C1—O2—Cu1	116.7 (3)	O4—C6—O3	126.2 (4)
C6—O3—Cu1^iii^	118.9 (3)	O4—C6—C3	117.2 (4)
C6—O3—Cu1^iv^	137.1 (3)	O3—C6—C3	116.4 (4)
Cu1^iii^—O3—Cu1^iv^	103.25 (12)	O5—C7—N3	125.4 (5)
C7—O5—Cu1	122.7 (3)	O5—C7—H7	117.3
H6A—O6—H6B	113.5 (19)	N3—C7—H7	117.3
C5—N1—C2	118.1 (4)	N3—C8—H8A	109.5
C5—N1—Cu1	129.7 (3)	N3—C8—H8B	109.5
C2—N1—Cu1	112.2 (3)	H8A—C8—H8B	109.5
C4—N2—C3	116.4 (4)	N3—C8—H8C	109.5
C7—N3—C9	120.4 (5)	H8A—C8—H8C	109.5
C7—N3—C8	121.9 (5)	H8B—C8—H8C	109.5
C9—N3—C8	117.6 (5)	N3—C9—H9A	109.5
O1—C1—O2	124.5 (4)	N3—C9—H9B	109.5
O1—C1—C2	120.4 (4)	H9A—C9—H9B	109.5
O2—C1—C2	115.1 (3)	N3—C9—H9C	109.5
N1—C2—C3	121.0 (4)	H9A—C9—H9C	109.5
N1—C2—C1	113.7 (3)	H9B—C9—H9C	109.5
			
O5—Cu1—O2—C1	−169.1 (3)	O1—C1—C2—C3	3.7 (7)
O3^i^—Cu1—O2—C1	71 (3)	O2—C1—C2—C3	−175.6 (4)
N1—Cu1—O2—C1	2.3 (3)	C4—N2—C3—C2	−0.4 (6)
O3^ii^—Cu1—O2—C1	95.5 (3)	C4—N2—C3—C6	177.3 (4)
O2—Cu1—O5—C7	−11.7 (4)	N1—C2—C3—N2	1.5 (6)
O3^i^—Cu1—O5—C7	166.0 (4)	C1—C2—C3—N2	−178.8 (4)
N1—Cu1—O5—C7	−64.7 (9)	N1—C2—C3—C6	−176.0 (4)
O3^ii^—Cu1—O5—C7	89.4 (4)	C1—C2—C3—C6	3.7 (7)
O2—Cu1—N1—C5	179.2 (4)	C3—N2—C4—C5	−0.5 (6)
O5—Cu1—N1—C5	−127.0 (7)	C2—N1—C5—C4	0.8 (6)
O3^i^—Cu1—N1—C5	1.7 (4)	Cu1—N1—C5—C4	−178.4 (3)
O3^ii^—Cu1—N1—C5	78.9 (4)	N2—C4—C5—N1	0.3 (7)
O2—Cu1—N1—C2	0.1 (3)	Cu1^iii^—O3—C6—O4	7.2 (6)
O5—Cu1—N1—C2	53.8 (8)	Cu1^iv^—O3—C6—O4	175.4 (3)
O3^i^—Cu1—N1—C2	−177.5 (3)	Cu1^iii^—O3—C6—C3	−167.7 (3)
O3^ii^—Cu1—N1—C2	−100.3 (3)	Cu1^iv^—O3—C6—C3	0.5 (6)
Cu1—O2—C1—O1	176.6 (4)	N2—C3—C6—O4	−84.7 (5)
Cu1—O2—C1—C2	−4.1 (5)	C2—C3—C6—O4	92.9 (5)
C5—N1—C2—C3	−1.6 (6)	N2—C3—C6—O3	90.7 (4)
Cu1—N1—C2—C3	177.6 (3)	C2—C3—C6—O3	−91.7 (5)
C5—N1—C2—C1	178.7 (4)	Cu1—O5—C7—N3	179.4 (4)
Cu1—N1—C2—C1	−2.1 (4)	C9—N3—C7—O5	0.6 (9)
O1—C1—C2—N1	−176.6 (4)	C8—N3—C7—O5	179.3 (6)
O2—C1—C2—N1	4.1 (5)		

Symmetry codes: (i) −*x*+1, *y*−1/2, −*z*+1/2; (ii) *x*, −*y*+3/2, *z*−1/2; (iii) −*x*+1, *y*+1/2, −*z*+1/2; (iv) *x*, −*y*+3/2, *z*+1/2.

### Hydrogen-bond geometry (Å, °)

**Table:**

*D*—H···*A*	*D*—H	H···*A*	*D*···*A*	*D*—H···*A*
O6—H6B···O4^v^	0.82 (5)	2.00 (3)	2.771 (5)	156 (7)
O6—H6A···O1^vi^	0.82 (5)	2.38 (3)	3.138 (6)	153 (5)
O6—H6A···O2^vi^	0.82 (5)	2.30 (3)	3.037 (5)	149 (5)

Symmetry codes: (v) *x*, *y*−1, *z*; (vi) −*x*+1, −*y*+1, −*z*.
